# Complications of grafts used in female pelvic floor reconstruction: Mesh erosion and extrusion

**DOI:** 10.4103/0970-1591.32067

**Published:** 2007

**Authors:** Tanya M. Nazemi, Kathleen C. Kobashi

**Affiliations:** Continence Center at Virginia Mason Medical Center, Seattle, Washington, USA

**Keywords:** Biomaterials, erosion, management, synthetic mesh

## Abstract

**Introduction:**

Various grafts have been used in the treatment of urinary incontinence and pelvic prolapse. Autologous materials such as muscle and fascia were first utilized to provide additional anatomic support to the periurethral and pelvic tissues; however, attempts to minimize the invasiveness of the procedures have led to the use of synthetic materials. Complications such as infection and erosion or extrusion associated with these materials may be troublesome to manage. We review the literature and describe a brief overview of grafts used in pelvic floor reconstruction and focus on the management complications specifically related to synthetic materials.

**Materials and Methods:**

We performed a comprehensive review of the literature on grafts used in pelvic floor surgery using MEDLINE and resources cited in those peer-reviewed manuscripts. The results are presented.

**Results:**

Biologic materials provide adequate cure rates but have associated downfalls including potential complications from harvesting, variable tissue quality and cost. The use of synthetic materials as an alternative graft in pelvic floor repairs has become a popular option. Of all synthetic materials, the type I macroporous polypropylene meshes have demonstrated superiority in terms of efficacy and fewer complication rates due to their structure and composition. Erosion and extrusion of mesh are common and troublesome complications that may be managed conservatively with observation with or without local hormone therapy, with transvaginal debridement or with surgical exploration and total mesh excision, dependent upon the location of the mesh and the mesh type utilized.

**Conclusions:**

The ideal graft would provide structural integrity and durability with minimal adverse reaction by the host tissue. Biologic materials in general tend to have fewer associated complications, however, the risks of harvesting, variable integrity of allografts, availability and high cost has led to the development and use of synthetic grafts. Synthetic grafts have a tendency to cause higher rates of erosion and extrusion; however, these complications can be managed successfully.

The use of graft materials in pelvic floor reconstruction is now a common practice. The evolution of grafts from autologous muscle and fascia has produced new materials that will hopefully prove to be efficacious and durable. This review aims to provide an overview of the different materials available for use in pelvic floor surgery and to discuss the potential complications, in particular erosion and extrusion, associated with synthetic materials.

## GRAFT MATERIAL

The decision to use a graft in the repair of the pelvic floor is based on a number of factors including the tissue quality of the patient, history of previous repairs and concomitant procedures to be performed. The ideal material should be strong, sterile, permanent, nonallergenic, inert, free of risk of infection, infectious transmission, erosion and extrusion and affordable.[1] In 1997, the female stress urinary incontinence clinical guidelines panel performed a meta-analysis of the literature from 1994-1997 and found an overall vaginal extrusion rate of 0.0001% vs. 0.007% for autologous and synthetic materials, respectively. Similarly, urethral erosion rates were noted in 0.003% and 0.02%, respectively.[2]

## BIOLOGIC MATERIALS

Graft materials may be categorized as biologic or synthetic. Biologic materials include autologous grafts, allografts and xenografts [Table 1]. Biografts were initially used for their histological similarity to human tissue, *in vivo* tissue remodeling and reduced erosion rates.[3] Specific advantages and disadvantages are noted with each type of graft material.

**Table 1:** Biologic materials used in pelvic floor reconstruction

Biologic material	Source	Trade Name
Autologous graft	Fascia lata	
	Rectus fascia	
Allograft	Human dermis	Alloderm (LifeCell, Branchburg, NJ)
		Bard^®^ Dermal Allograft (CR Bard, Haverhill, Rl)
		Axis™ Tutoplast^®^ Processed Dermis (Mentor Corp, Santa Barbara, CA)
		Repliform^®^ Tissue Regeneration Matrix (Boston scientific, Batick, MA)
	Human fascia lata	Suspend^®^ Tutoplast^®^ Processed Fascia Lata (Mentor Corp, Santa Barbara, CA)
		FasLata^®^ Allograft (CR Bard, Haverhill, Rl)
	Human dura mater	Lyodura (B Braun Melsungen AG, Germany)
Xenograft	Porcine dermis	Pelvicol^®^, Pelvilace^®^ (CR Bard, Haverhill, Rl)
		InteXen (American medical systems, Minnetonka, MN)
	Bovine dermis	Xenform™ Soft Tissue Repair Matrix (Boston Scientific, Natick, MA)
	Porcine small intestine submucosa	Surgisis^®^, Stratasis^®^ (Cook urological, Spencer, IN)

### Autologous grafts

Autologous grafts that are commonly harvested for repairs are rectus fascia and fascia lata. Clear advantages to using the patient's own tissue are decreased risk of erosion, rejection and infection. Since autologous fascia was one of the original graft materials utilized in pelvic floor repair, there is longer term data to suggest that the grafts provide durable results. A recent retrospective review was performed on 303 women who underwent autologous and cadaveric grafts over a nine-year period with a minimum 12-month follow-up. The results showed higher rates of recurrent leakage and reoperation for stress urinary incontinence (39.6% vs. 28.3% and 12.7% vs. 3.3%, respectively) following the use of cadaveric versus autologous fascia.[4] However, the use of autologous materials was associated with increased pain, risk of hernia formation at the harvesting site and increased operative time. Nevertheless, both allografts and autografts can be of variable quality depending on the patient's age and associated medical conditions.

### Allografts

Because of the potential morbidity associated with harvesting autologous fascia, the use of allograft tissue can be a desirable alternative. Human tissue procured from cadavers is harvested within 24h of death and is cultured and processed to reduce potential risk of disease transmission. The graft materials most commonly used include cadaveric fascia lata and dermis. Processing techniques to sterilize and prepare these tissues include irradiation, freeze-drying or solvent dehydration. The different processing techniques can affect the integrity of the grafts, which is a potential disadvantage with cadaveric materials; however, the discussion of the biomechanics of the allograft tissues is beyond the scope of this manuscript. Despite these techniques, there is still a remote risk of 1/1,667,600 for transmission of human immunodeficiency virus and a theoretical risk of prion transmission.[5,6] Historically used materials such as the lyophilized, irradiated human dura mater Lyodura (B Braun Melsungen AG, Germany) were associated with higher rates of transmission of the Creutzfeldt-Jakob disease. Other disadvantages to using these materials include availability and cost. Nevertheless, the overall reported success for allografts in pelvic repairs has been good (84-98%) with low extrusion rates.[7,8]

Amundsen *et al* reviewed 104 patients who underwent placement of allograft fascia lata pubovaginal slings with 19.4 ± 10.3 month follow-up. No vaginal extrusions or urinary tract erosions were noted.[7] Another review of 69 patients who underwent pelvic repair using dermal allograft material was performed by Drake *et al* who noted a 10.9% vaginal extrusion rate. All cases were managed expectantly and with vaginal estrogen cream and all resolved spontaneously.[9] Others, including the authors, have had favorable experience with conservative management of cadaveric fascial extrusion and do not believe that formal surgical excision is generally necessary. This review does not provide an exhaustive analysis of all biograft studies; however, Table 2 lists the extrusion rates reported in some allograft series.

**Table 2:** Erosion/extrusion rates for various al log rafts[9,39–41]

Graft	Study	No. patients (repair)	No. Erosion/extrusion (%)	Description of erosion/extrusion	Management
Dermal allograft	Clemons *et al* (2003)	33 (anterior)	0 (0%)		
	Drake *et al* (2005)	69 (21 anterior, 45 posterior, 3 both)	7 (10.9%)	Vaginal extrusion (3 anteriorly, 4 posteriorly)	Conservative with topical estrogen cream. All experienced spontaneous resolution
Allograft fascia lata	Flynn *et al* (2005)	24 (sacrocolpopexy)	0 (0%)		
	Frederick *et al* (2005)	251 (cadaveric prolapse repair with	22 (9%) sling (CaPS)	Intravaginal granulation tissue caused by extrusion of panacryl sutures used for the cystocele repair and vault suspension	Patients treated by suture removal and fulguration of the granulation tissue with silver nitrate

### Xenografts

Xenografts such as porcine dermis and small intestinal submucosa (SIS) provide other biograft alternatives. These materials offer potential advantages over allografts in that they are more readily available and there is no theoretical risk of human viral transmission. Porcine SIS and dermis are processed to remove cellular components, leaving only collagen and elastin fibers that do not elicit an immune response. This allows for remodeling of the sling by host tissue.[10–12] Two separate prospective randomized studies comparing porcine dermal pubovaginal sling to the tension-free vaginal tape (TVT), which utilizes a synthetic polypropylene mesh, showed similar cure rates of 89% and 85% at a median follow-up of 12 months and 82.4% and 88.3% at a median follow-up of 36 months.[13,14] Xenografts are costly and long-term data is still lacking. Table 3 lists the success and complications associated with various xenograft series.

**Table 3:** Success and complication rates of various xenografts[11,12,42–44]

Graft	Study	No. patients (repair)	Cure rate	Complications
Porcine dermis	Gomelsky *et al* (2003)	70 (cystocele)	91%	12.9% recurrent cystocele at a mean follow-up of 24 months
	Giri *et al* (2006)	48 (pubovaginal sling)	54%	1 urethrolysis, 1 suprapubic wound infection, 1 urinary tract infection, 2 vaginal bleeding, 2 pain during intercourse, 2 deep pelvic pain
Porcine small intestinal submucosa	Jones *et al* (2005)	34 (mid-urethral sling)	79%	9% developed suprapubic inflammation
	Rutner *et al* (2003)	152 (pubovaginal sling with bone anchors)	93.4%	4.6% recurrent stress urinary incontinence
	Ho *et al* (2003)	10 (pubourethral sling)	90%	60% - six patients presented with postoperative inflammatory reactions

## SYNTHETIC MATERIALS

Synthetic materials may have some advantages over biologic materials in terms of disease transmission, durability, tensile strength and availability.[10] The types of mesh are categorized based on pore size and fiber type as originally described for their use in abdominal wall hernia repairs.[15] While the advantages of using synthetics for vaginal surgery are evident, there are specific concerns regarding their use. This includes complications associated with the surgical procedure itself such as bleeding, hematoma formation, bladder and bowel injury, adhesions and obstructive ileus and complications from the material itself, including infection, urinary tract erosion and vaginal extrusion, sinus-tract formation and abscess formation. In addition, functional problems may arise including *de novo* urgency, urge incontinence and urinary.[16,17] Table 4 lists the properties of the various synthetic materials available and their use in pelvic reconstruction.

**Table 4:** Properties of synthetic materials

Mesh type	Pore size	Structure	Synthetic material	Trade name	Use in pelvic floor reconstruction
I	>75 μm	Monofilament	Polypropylene	Uretex^®^ Self-Anchoring Urethral Support System (CR Bard, Haverhill, Rl)	Transvaginal
				Uretex^®^ TO Trans-Obturator Urethral Support System (CR Bard, Haverhill, Rl)	Transobturator
				Gynecare TVT (Ethicon/Johnson and Johnson, Somerville, NJ)	Transvaginal
			Somerville, NJ)	Gynecare TVT-O (Ethicon/Johnson and Johnson, transobturator	Inside-out
				SPARC™ Self-fixating Sling System (American Medical Systems, Minnetonka, MN)	Suprapubic
				In-Fast™ Ultra Transvaginal Sling (American Medical Systems, Minnetonka, MN)	Transvaginal with bone anchors
				Monarc™ Subfascial Hammock (American Medical Systems, Minnetonka, MN)	Transobturator
				Lynx^®^ Suprapubic Mid-Urethral Sling System (Boston Scientific, Natick, MA)	Suprapubic
				Advantage^®^ Transvaginal Mid-Urethral Slinj System (Boston Scientific, Natick, MA)	Transvaginal
				Obtryx^®^ Transobturator Mid-Urethrai Sling System (Boston Scientific, Natick, MA)	Transobturator
				T-Sling (Caldera Medical, Augurr. H ms, CA)	Suprapubic, transvaginal or transobturator approach
				Aris™ Trans-obturator Tape (Mentor Corp, Santa Natick, CA)	Transobturator
				Perigee™ (American Medial Systems. Minnetonka, MN)	Transobturator anterior prolapse repair
				Apogee™ (American Medial Systems, Minnetonkc, MN)	Transvaginal vaginal vault prolapse repair
				Gynecare Prolift (Ethicon/Johnson and Johnson, Somerville, NJ)	Transvaginal vaginal vault prolapse repair
				Prolene (Ethicon/joimson and Johnson, Somerville, NJ)	Variable use
				Atrium (Atiiun Medical, Hudson, NH)	Variable use
				Marlex^®^ (CR Bard, Cranston, RI)	Variable use
II	< 10 μm	Multifilament	Expanded PTFF	Gore-Tex^®^ (W. L. Gore, Flagstaff, AZ)	Variable use
III	< 10 μm (macroporous with microporous components)	Multifilament	PTFE	Teflon (CR Bard, Haverhill, RI)	Sacrocolpopexy, suprapubic, transvaginal
			Polyethylene terepthalate	Mersilene (Ethicon/Johnson and Johnson, Somerville, NJ)	Sacrocolpopexy, suprapubic, transvaginal
			Polypropylene	IVS Tunneller™ (Tyco Healthcare, Norwalk, CT)	Transvaginal
				Obturator IVS Tunneller™ (Tyco Healthcare, Norwalk, CT)	Transobturator
			Woven polyester	ProteGen (Boston Scientific, Natick, MA)	Recalled 1999
IV	<1 μm	Multifilament	Silicone-coated polyester	Intemesh (American Medical Systems, Minnetonka, MN)	Sacrocolpopexy, suprapubic, transvaginal
			Dura mater substitute	PRECLUDE^®^ MVP^®^ Dura Substitute (W. L. Gore, Flagstaff, AZ)	
			Expanded PTFE, pericardial membrane substitute	PRECLUDE^®^ Pericardial Membrane (W. L. Gore, Flagstaff, AZ)	

Adapted from Karlovsky *et al* 2005.[6]

### Type I mesh

Type I meshes have a pore size of >75 mm, which is considered macroporous. They are composed of polypropylene monofilaments. The large pores allow access for leukocytes and macrophages, as well as ingrowth of fibroblasts and collagen and neovascularization. All this contributes to lower infection rates and promotes tissue incorporation into the host.

### Type II mesh

Type II meshes have a pore size of <10 mm and include a multifilament expanded polytetrafluoroethylene mesh that only allows passage of histiocytes. There is therefore minimal incorporation into host tissue.

### Type III mesh

Type III meshes are braided or multifilamentous with both macroporous and microporous components.

### Type IV mesh

Type IV meshes have a submicronic pore size of <1 mm. Due to the sheet-like material that has poor tissue incorporation, this mesh is not often used in vaginal reconstruction. One exception is the polyethylene terephthalate fabric coated with silicone that has large pores with some submicronic components as well.[18]

## EROSION AND EXTRUSION

For the purpose of this paper, we define “erosion” as the presence of graft material in the lumen of the urinary tract and “extrusion” as the presence of exposed graft material in the vagina.

Use of Type I mesh has demonstrated consistent success with similar rates of vaginal extrusion regardless of the technique for placement. Extrusion rates of 0.4-4.8% for TVT, 1-10.5% for SPARC and 0-6.7% for the transobturator Monarc have been reported.[19–23] A second transobturator sling that utilized a fusion-welded, thermally bonded, nonwoven, nonknitted polypropylene mesh (Ob-Tape™, Mentor Corp, Santa Barbara, CA) had significantly higher rates of extrusion ranging from 10-20%.[22,24–26]

Comparable extrusion rates of 0-19% have been reported with sacrocolpopexy.[27,28] New techniques (Apogee™ and Perigee™ (American Medical Systems, Minnetonka, MN) and Gynecare Prolift (Ethicon, Sommerville, NJ) have been developed that place polypropylene via the transobturator or transvaginal approach in the repair of anterior vaginal wall prolapse or vaginal vault prolapse. These techniques have shown promising early results, but intermediate and long-term data on rates of erosion and extrusion are yet to be presented.[29,30]

**Figure 1:**
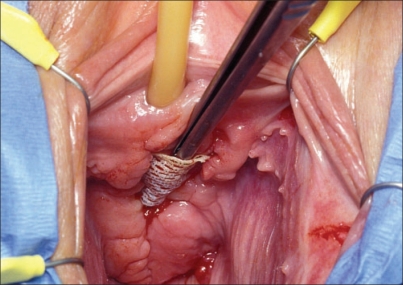
Vaginal extrusion of type IV silicone mesh. Note the lack of tissue incorporation and granulation

Type II and III meshes are multifilamentous and therefore may allow bacteria to pass through and adhere to the graft and surrounding tissues. The small pore size does not allow passage of macrophages and leukocytes that may counter invading bacteria. It is because of these theoretical issues that Type II and III meshes are now rarely, if ever, used in pelvic floor reconstruction. Similarly, Type IV mesh has pore sizes too small to allow for fibroblast and leukocyte infiltration. They tend to induce pseudocapsules that may harbor infection. High rates of erosion, extrusion and other complications were noted and subsequently, Type IV mesh is rarely used in pelvic reconstructive surgery [Figure 1].[18] Table 5 lists reported extrusion and erosion rates for Type II, III and IV meshes.

**Table 5:** Erosion/extrusion rates for various synthetic meshes[27,28,38,45–51]

Mesh type	Material	Trade name	Study	No. patients	No. erosion/extrusion (%)	Description of complication
II	Expanded PTFE	Gore-Tex^®^ W. L. Gore, Flagstaff, AZ)	Choe *et al* (1999)	90	5 (5.6)	Vaginal granulation requiring removal of mesh
			Begley *et al* (2005)	33	3 (9)	Vaginal extrusion
			Weinberger *et al* (1995)	98	25 (26)	Ten vaginal extrusions, ten granulation tissue, five sinus tracts
III	PTFE	Teflon (CR Bard, Haverhill, RI)	Yamada *et al* (2001)	137	1 (0.7)	Urethral erosion
			Nygaard *et al* (2004)	119	6 (5.5)	Mesh erosion or extrusion following sacrocolpopexy
	Polyethylene terephthalate	Mersilene (Ethicon/Johnson and Johnson, Somerville, NJ)	Young *et al* (2001)	176	8 (4)	Seven vaginal and one inguinal sling extrusion
			Kohli *et al* (1998)	10	2 (20)	Vaginal extrusion
	Polypropylene	IVS Tunneller™ (Tyco Healthcare, Norwalk, CT)	Siegel *et al* (2005)	35	6 (17%)	Vaginal extrusion
	Woven polyester	ProteGen (Boston Scientific, Natick, MA) - recalled 1999	Kobashi *et al* (1999)	N/A	34	vaginal extrusion, infection or pain all requiring removal
IV	Silicone-coated polyester	Intemesh (American Medical Systems,	Begley *et al* (2005)	21	4 (19)	Vaginal extrusion
		Minnetonka, MN)	Duckett *et al* (2000)	7	5 (71)	vaginal extrusion and sinus formation

## RISK FACTORS

As discussed previously, erosion or extrusion of the mesh is thought to be associated with the type of synthetic material used. However, patient factors such as poorly controlled diabetes mellitus, tobacco use, prior history of pelvic irradiation, repeat procedures and vaginal estrogen status may also contribute to poor wound healing and subsequent infection, erosion or extrusion. Some studies have suggested that concomitant hysterectomy may be an additional risk factor for extrusion of the sacrocolpopexy mesh.[28,31] Surgical techniques such as excessive tension and unrecognized urethral or vesical injury may also contribute to higher rates of urinary tract erosion.[32] In addition, rolling of the tape during placement or vaginal suturing may produce a narrow band that can result in pressure necrosis and erosion.[20]

While repairs requiring greater dissection tend to have higher rates of complications, the placement of slings via the transvaginal versus transobturator route do not appear to play a significant role in the risk of erosion or extrusion in the literature available to date. Prospective randomized trials that compare sling placement techniques are currently in progress.

## DIAGNOSIS AND MANAGEMENT

Patients who present with vaginal extrusion or urinary tract erosion may demonstrate a variety of symptoms, but they may be completely asymptomatic. Usual presenting symptoms include vaginal discharge, pain, dyspareunia, complaints of pain from the partner during intercourse, *de novo* stress urinary incontinence, urgency, hematuria or urinary tract infection or obstruction. In the experience of the authors, physical exam findings can usually identify extrusion of mesh components on pelvic exam. However, in cases of high suspicion without visualization of extruded mesh, exam under anesthesia may be necessary. It is of utmost importance to evaluate the urinary tract with cystourethroscopy to rule out erosion of material into the bladder or urethra, particularly if the patient presents with hematuria, recurrent urinary tract infections, irritative or obstructive symptoms, *de novo* urgency or bladder stones. In addition, we have noted from our own experience that over 30% of patients with vaginal extrusions required exam under anesthesia in order to adequately identify their extrusion sites, demonstrating the importance of a high index of suspicion for extrusion in those with clinical indications.

Management is based on the type of material, presence of infection and location of erosion or extrusion. From our own experience and from review of the literature, we have found that extrusion of Type I polypropylene mesh into the vagina may be managed conservatively with abstinence from sexual intercourse, local estrogen replacement therapy and antibiotics if associated infection is noted.[1,33] The clinician should counsel the patient on the possible length of healing time (six to eight weeks) as some patients may prefer to proceed with definitive treatment rather than abstaining from intercourse for this length of time. Spontaneous healing rates from 29-100% have been reported with conservative management.[1,33] One group described 26% cure rate with abstinence and local vaginal antiseptics for one month.[31] If these measures fail after six to eight weeks, then excision of the exposed mesh with adequate debridement of underlying and surrounding tissues will allow for improved wound healing.

**Figure 2:**
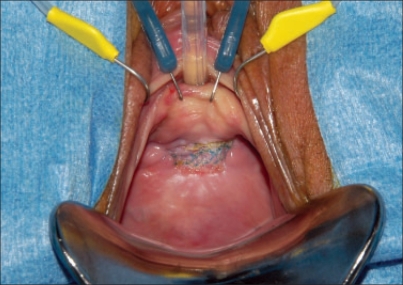
Vaginal extrusion of type I polypropylene mesh

Persistent infection or failure to epithelialize over a type I mesh warrants complete mesh excision [Figure 2]. The approach to removal of the mesh is often mandated by the location of the mesh. In general, pubovaginal, transobturator and transvaginal slings may be removed transvaginally and mesh placed for sacrocolpopexy should be removed abdominally. If complete removal of extruded mesh via the transvaginal route is not feasible, then transabdominal approach may be indicated for the retropubic slings. Transobturator slings, when associated with infection, may require exploration of the thigh in extreme cases.

Vaginal extrusion of type II, III and IV meshes generally requires complete excision due to higher risks of infection and poor healing rates. As with type I mesh, excision may be performed transvaginally if the exposed graft can be removed completely with adequate debridement and reapproximation of vaginal epithelium.[28] Ironically, the mesh types that induce pseudocapsule formation tend to be easier to remove than the type I meshes that allow extensive tissue regrowth.

**Figure 3:**
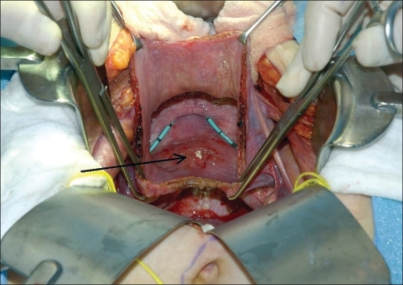
Bladder erosion of type I polypropylene mesh following vaginal vault suspension

Erosion into the urinary tract mandates complete removal of mesh regardless of mesh type [Figure 3]. Erosion of mesh into the bladder is rare and has traditionally been excised using a transvesical approach. Patients often present with hematuria, irritative voiding symptoms, urinary tract infection or retention. Cystoscopic resection of intravesical materials has been reported by Clemens *et al*. Of 14 patients that presented with complications following pubovaginal sling placement, two were noted to have erosion of mesh into the bladder and both were managed by endoscopic sling and/or suture removal. At one month follow-up, both patients' symptoms had resolved and both were continent.[34] One must use caution to remove as much mesh as possible when using a cystoscopic approach as the retained mesh may continue to erode and potentiate symptoms.

Urinary tract erosion of mesh that has been used in sacrocolpopexy may be addressed via laparoscopy or laparotomy with retroperitoneal graft excision.

Urethral erosions require urethrolysis with graft explantation. Urethral debridement followed by primary repair and multilayer closer with a Martius flap has been described by Amundsen *et al*. In a review of nine patients who presented with erosion of graft material into the urethra, three were from synthetic grafts that were excised and repaired as described. At a mean follow-up of 30 months, no urethral erosion or fistulas occurred, however, stress incontinence recurred in two of the three patients.[32] Clemens *et al* recommend urethral catheter drainage for two weeks with a pull-out cystourethrogram at the time of catheter removal.[34]

Newer techniques have been described in the treatment of mesh extrusion and erosion. Laparoscopic excision of mesh associated with bladder erosion and transvaginal endoscopic removal of mesh after sacrocolpopexy have been described.[35,36] Pikaart *et al* report three patients with mesh noted in the bladder following pubovaginal placement of polypropylene mesh tape. All three underwent successful laparoscopic removal of the mesh and at six months follow-up, two of the three patients continued to have complaints of stress and urge incontinence without further mesh erosion into the bladder.[36] Romero *et al* describe three cases of vaginal mesh extrusion following abdominal sacrocolpopexy treated by transvaginal endoscopic excision of mesh. All three patients continued to have excellent support with adequate tissue healing at follow-up (six weeks to one year).[35] A technique of cystoscopic excision using a suprapubic port to excise the mesh has also been described by Rosenblatt *et al*. Two cases of bladder erosion following TVT were managed using cystoscopic excision combined with traction from laparoscopic grapsers through a small suprapubic port. One of the patients had follow-up office cystoscopy at six weeks that showed appropriate resolution with no persistent mesh in the bladder.[37]

Continence rates following mesh removal have been variable and often dependent on the amount of dissection performed and presence of infection. Reported rates of continence have ranged from 42-100%.[20,34,38]

## CONCLUSIONS

Synthetic mesh has become a popular option for pelvic reconstruction. The potential complications of urinary tract erosion and vaginal extrusion are dependent on multiple factors including mesh type and patient tissue integrity. However, review of short and intermediate term data from the literature has shown that amongst synthetic grafts, type I mesh provides durable results with the fewest rates of erosion and extrusion. In addition, viable management options for vaginal extrusion include conservative approaches such as observation with or without local estrogen administration. While all materials, synthetic and biologic alike, have advantages and disadvantages in the treatment of pelvic floor disorders, synthetic materials may provide a safe and cost-effective alternative for pelvic reconstructive surgery.

## References

[1] Kobashi KC, Govier FE (2003). Management of vaginal erosion of polypropylene mesh slings. J Urol.

[2] Leach GE, Dmochowski RR, Appell RA, Blaivas JG, Hadley HR, Luber KM (1997). Female Stress Urinary Incontinence Clinical Guidelines Panel summary report on surgical management of female stress urinary incontinence. The American Urological Association. J Urol.

[3] Silva WA, Karram MM (2005). Scientific basis for use of grafts during vaginal reconstructive procedures. Curr Opin Obstet Gynecol.

[4] Howden NS, Zyczynski HM, Moalli PA, Sagan ER, Meyn LA, Weber AM (2006). Comparison of autologous rectus fascia and cadaveric fascia in pubovaginal sling continence outcomes. Am J Obstet Gynecol.

[5] Wilson TS, Lemack GE, Zimmern PE (2003). Management of intrinsic sphincteric deficiency in women. J Urol.

[6] Karlovsky ME, Thakre AA, Rastinehad A, Kushner L, Badlani GH (2005). Biomaterials for pelvic floor reconstruction. Urology.

[7] Amundsen CL, Visco AG, Ruiz H, Webster GD (2000). Outcome in 104 pubovaginal slings using freeze-dried allograft fascia lata from a single tissue bank. Urology.

[8] Wright EJ, Iselin CE, Carr LK, Webster GD (1998). Pubovaginal sling using cadaveric allograft fascia for the treatment of intrinsic sphincter deficiency. J Urol.

[9] Drake NL, Weidner AC, Webster GD, Amundsen CL (2005). Patient characteristics and management of dermal allograft extrusions. Int Urogynecol J Pelvic Floor Dysfunct.

[10] Amrute KV, Badlani GH (2006). Female incontinence: A review of biomaterials and minimally invasive techniques. Curr Opin Urol.

[11] Jones JS, Rackley RR, Berglund R, Abdelmalak JB, DeOrco G, Vasavada SP (2005). Porcine small intestinal submucosa as a percutaneous mid-urethral sling: 2-year results. BJU Int.

[12] Gomelsky A, Rudy DC, Dmochowski RR (2004). Porcine dermis interposition graft for repair of high grade anterior compartment defects with or without concomitant pelvic organ prolapse procedures. J Urol.

[13] Abdel-Fattah M, Barrington JW, Arunkalaivanan AS (2004). Pelvicol pubovaginal sling versus tension-free vaginal tape for treatment of urodynamic stress incontinence: A prospective randomized three-year follow-up study. Eur Urol.

[14] Arunkalaivanan AS, Barrington JW (2003). Randomized trial of porcine dermal sling (Pelvicol implant) vs. tension-free vaginal tape (TVT) in the surgical treatment of stress incontinence: A questionnaire-based study. Int Urogynecol J Pelvic Floor Dysfunct.

[15] Amid PK, Shulman AG, Lichtenstein IL, Hakakha M (1994). Biomaterials for abdominal wall hernia surgery and principles of their applications. Langenbecks Arch Chir.

[16] Baessler K, Maher CF (2006). Mesh augmentation during pelvic-floor reconstructive surgery: Risks and benefits. Curr Opin Obstet Gynecol.

[17] Bhargava S, Chapple CR (2004). Rising awareness of the complications of synthetic slings. Curr Opin Urol.

[18] Comiter CV, Colegrove PM (2004). High rate of vaginal extrusion of silicone-coated polyester sling. Urology.

[19] Abouassaly R, Steinberg JR, Lemieux M, Marois C, Gilchrist LI, Bourque JL (2004). Complications of tension-free vaginal tape surgery: A multi-institutional review. BJU Int.

[20] Huang KH, Kung FT, Liang HM, Chang SY (2005). Management of polypropylene mesh erosion after intravaginal midurethral sling operation for female stress urinary incontinence. Int Urogynecol J Pelvic Floor Dysfunct.

[21] Lord HE, Taylor JD, Finn JC, Tsokos N, Jeffery JT, Atherton MJ (2006). A randomized controlled equivalence trial of short-term complications and efficacy of tension-free vaginal tape and suprapubic urethral support sling for treating stress incontinence. BJU Int.

[22] Yamada BS, Govier FE, Stefanovic KB, Kobashi KC (2006). High rate of vaginal erosions associated with the mentor ObTape. J Urol.

[23] But I (2005). Vaginal wall erosion after transobturator tape procedure. Int Urogynecol J Pelvic Floor Dysfunct.

[24] Domingo S, Alama P, Ruiz N, Perales A, Pellicer A (2005). Diagnosis, management and prognosis of vaginal erosion after transobturator suburethral tape procedure using a nonwoven thermally bonded polypropylene mesh. J Urol.

[25] Siegel AL (2005). Vaginal mesh extrusion associated with use of Mentor transobturator sling. Urology.

[26] Robert M, Murphy M, Birch C, Swaby C, Ross S (2006). Five cases of tape erosion after transobturator surgery for urinary incontinence. Obstet Gynecol.

[27] Nygaard IE, McCreery R, Brubaker L, Connolly A, Cundiff G, Weber AM (2004). Abdominal sacrocolpopexy: A comprehensive review. Obstet Gynecol.

[28] Begley JS, Kupferman SP, Kuznetsov DD, Kobashi KC, Govier FE, McGonigle KF (2005). Incidence and management of abdominal sacrocolpopexy mesh erosions. Am J Obstet Gynecol.

[29] Palma P, Riccetto C, Dambros M, Netto NR (2006). New trends in the transobturator management of cystoceles. BJU Int.

[30] Reisenauer C, Kirschniak A, Drews U, Wallwiener D (2006). Transobturator vaginal tape inside-out. A minimally invasive treatment of stress urinary incontinence: Surgical procedure and anatomical conditions. Eur J Obstet Gynecol Reprod Biol.

[31] Collinet P, Belot F, Debodinance P, Ha Duc E, Lucot JP, Cosson M (2006). Transvaginal mesh technique for pelvic organ prolapse repair: Mesh exposure management and risk factors. Int Urogynecol J Pelvic Floor Dysfunct.

[32] Amundsen CL, Flynn BJ, Webster GD (2003). Urethral erosion after synthetic and nonsynthetic pubovaginal slings: Differences in management and continence outcome. J Urol.

[33] Achtari C, Hiscock R, O'Reilly BA, Schierlitz L, Dwyer PL (2005). Risk factors for mesh erosion after transvaginal surgery using polypropylene (Atrium) or composite polypropylene/polyglactin 910 (Vypro II) mesh. Int Urogynecol J Pelvic Floor Dysfunct.

[34] Clemens JQ, DeLancey JO, Faerber GJ, Westney OL, Mcguire EJ (2000). Urinary tract erosions after synthetic pubovaginal slings: Diagnosis and management strategy. Urology.

[35] Romero AA, Amundsen CL, Weidner AC, Webster GD (2004). Transvaginal endoscopic removal of eroded mesh after abdominal sacral colpopexy. Obstet Gynecol.

[36] Pikaart DP, Miklos JR, Moore RD (2006). Laparoscopic removal of pubovaginal polypropylene tension-free tape slings. JSLS.

[37] Rosenblatt P, Pulliam S, Edwards R, Boyles SH (2005). Suprapubically assisted operative cystoscopy in the management of intravesical TVT synthetic mesh segments. Int Urogynecol J Pelvic Floor Dysfunct.

[38] Kobashi KC, Dmochowski R, Mee SL, Mostwin J, Nitti VW, Zimmern PE (1999). Erosion of woven polyester pubovaginal sling. J Urol.

[39] Clemons JL, Myers DL, Aguilar VC, Arya LA (2003). Vaginal paravaginal repair with an AlloDerm graft. Am J Obstet Gynecol.

[40] Flynn MK, Webster GD, Amundsen CL (2005). Abdominal sacral colpopexy with allograft fascia lata: One-year outcomes. Am J Obstet Gynecol.

[41] Frederick RW, Leach GE (2005). Cadaveric prolapse repair with sling: Intermediate outcomes with 6 months to 5 years of follow-up. J Urol.

[42] Rutner AB, Levine SR, Schmaelzle JF (2003). Processed porcine small intestine submucosa as a graft material for pubovaginal slings: Durability and results. Urology.

[43] Giri SK, Hickey JP, Sil D, Mabadeje O, Shaikh FM, Narasimhulu G (2006). The long-term results of pubovaginal sling surgery using acellular cross-linked porcine dermis in the treatment of urodynamic stress incontinence. J Urol.

[44] Ho KL, Witte MN, Bird ET (2004). 8-ply small intestinal submucosa tension-free sling: Spectrum of postoperative inflammation. J Urol.

[45] Choe JM, Staskin DR (1999). Gore-Tex patch sling: 7 years later. Urology.

[46] Weinberger MW, Ostergard DR (1995). Long-term clinical and urodynamic evaluation of the polytetrafluoroethylene suburethral sling for treatment of genuine stress incontinence. Obstet Gynecol.

[47] Yamada T, Kamata S, Nagahama K, Ichiyanagi N, Horiuchi S, Saitoh H (2001). Polytetrafluoroethylene patch sling for type 2 or type 3 stress urinary incontinence. Int J Urol.

[48] Young SB, Howard AE, Baker SP (2001). Mersilene mesh sling: Short- and long-term clinical and urodynamic outcomes. Am J Obstet Gynecol.

[49] Kohli N, Walsh PM, Roat TW, Karram MM (1998). Mesh erosion after abdominal sacrocolpopexy. Obstet Gynecol.

[50] Duckett JR, Constantine G (2000). Complications of silicone sling insertion for stress urinary incontinence. J Urol.

[51] Siegel AL, Kim M, Goldstein M, Levey S, Ilbeigi P (2005). High incidence of vaginal mesh extrusion using the intravaginal slingplasty sling. J Urol.

